# A qualitative study exploring the digital therapeutic alliance with fully automated smartphone apps

**DOI:** 10.1177/20552076241277712

**Published:** 2024-12-15

**Authors:** Rebecca Brotherdale, Katherine Berry, Sandra Bucci

**Affiliations:** 1 Division of Psychology and Mental Health, School of Health Sciences, Faculty of Biology, Medicine and Health, 5292 The University of Manchester, Manchester Academic Health Sciences Centre, Manchester, UK; 2 9022 Greater Manchester Mental Health NHS Foundation Trust, Manchester, UK

**Keywords:** Digital health, mHealth, mental health, qualitative, psychology

## Abstract

**Background:**

Digital mental health interventions are increasingly used to scale up access to mental health support, yet very few mental health apps are empirically supported, with high attrition rates. The therapeutic alliance between therapists and clients is a key factor in predicting psychological therapy retention and outcomes. Understanding how this concept translates to the digital context, the so-called digital therapeutic alliance (DTA), may help enhance retention and outcomes in digital mental health.

**Objectives:**

To explore and conceptualise the DTA with mental health interventions delivered by standalone apps. Specifically: (1) whether people with mental health difficulties form an alliance with mental health apps; (2) what constitutes the DTA; and (3) the similarities and differences across the traditional therapeutic alliance and DTA.

**Methods:**

Qualitative semi-structured interviews were conducted with 17 individuals utilising mental health apps. Interview topic examples included rationale for app support, goals around app use and perceived connection. Interviews were analysed using thematic analysis.

**Results:**

Five core themes were identified: (1) connection with ‘an other’; (2) accessibility; (3) choice and empowerment; (4) goals and expectations: having lower expectations than in-person support; and (5) safe place: non-judgemental, loyalty, trust.

**Conclusions:**

The study strengthens support for the concept of the DTA but adds to existing knowledge by suggesting that the DTA needs to be conceived differently to the traditional therapeutic alliance. App developers can enhance alliance by paying particular attention to the use of human voice features, validating statements, personalised interfaces, and multiple options for activities and strategies.

## Introduction

The global prevalence of mental health disorders is increasing.^1^ According to data from the Officetable for National Statistics,^2^ the proportion of people with depressive symptoms in the UK has doubled since the onset of the COVID-19 pandemic. Despite the urgent need for mental health care, there is significant disparity between those who require it and those who have access to it. The COVID-19 pandemic and the resultant disruptions in mental health care delivery, coupled with an escalation in service demand, intensified the existing challenges related to timely access to mental health services.^2^ Consequently, these circumstances catalysed the digital transformation of healthcare,^3^ with a noticeable surge in the development and evaluation of digital health interventions (DHIs). DHIs are defined as the delivery of health care through digital and mobile technologies.^4^ DHIs can enhance the availability and accessibility to evidence-based treatment^5^ and may assist in engaging hard-to-reach groups by offering greater anonymity and reducing stigma or discomfort in sharing mental health experiences compared to traditional in-person interventions.^6^ The widespread availability of smartphones has given rise to DHIs being delivered via smartphone applications (apps). Digital health apps include apps for self-monitoring symptoms^7^ and delivery of therapeutic techniques and strategies, such as mindfulness^8^ and cognitive behaviour therapy.^9^ Apps are increasingly used for treating mood and anxiety disorders, either with or without therapy assistance.^10–12^ DHIs have an emerging evidence base, with recent systematic reviews and meta-analyses demonstrating their effectiveness in improving various mental health difficulties, especially common mental health problems, such as anxiety and depression,^13,14^ but also in more severe mental problems such as psychosis.^9^ Despite the availability and popularity of mental health apps on app stores^15^ attracting several thousand monthly active users,^16^ mental health-based apps have high rates of attrition over time.^17,18^ Poor design and lack of personalisation are considered major contributing factors.^19^ Despite these rather consistent findings in the literature, the reasons for high attrition rates have not been well investigated from the user's perspective. In face-to-face therapy, high rates of attrition are associated with the therapeutic alliance (TA^20^).

The TA consistently predicts engagement and outcomes across therapeutic modalities.^21,22^ The TA typically refers to the relationship between the client and therapist, with Bordin's^23^ widely adopted conceptualisation of the TA as: (1) shared goals; (2) mutual agreement on tasks; and (3) the development of a bond, involving a level of trust. Bordin (1979) further described the alliance change process as ‘a person who seeks change and the one who offers to be the change agent,’ (p. 252). Several studies indicate the existence of the so-called ‘digital therapeutic alliance’ (DTA), a concept that applies the notion of the TA to online and digital therapies.^24^

A growing body of research has shown that the DTA can have a similar effect to in-person TA.^25^ Moreover, the DTA has been rated highly even in the absence of therapist contact,^26^ implying that the physical presence of a therapist is not necessary for establishing a DTA. However, it appears the DTA has a weaker association with therapeutic outcomes than the TA with in-person contact.^27^ Therefore, it is possible that the TA may play a different role in DHIs than with in-person interventions.

The idea that users can form a TA with fully automated DHIs is less intuitive than the conventional TA between humans. However, fully automated DHIs hold unique value due to their accessibility, and some users prefer unsupported (absence of therapist) DHIs because they perceive them as trustworthy, to be less judgemental than therapists, and feeling more comfortable being honest.^28,26^ Clarity around the DTA has also been considered a top ten research priority in the James Lind Alliance priority setting partnership.^29^ However, research into the DTA is nascent, with a small number of studies exploring the DTA with fully automated apps. One narrative review of five studies by Henson and colleagues^30^ concluded that the nature and significance of the DTA with apps remains ambiguous due to measurement challenges, especially where TA measures have simply adapted items to reflect the digital context. Another review^31^ that examined the DTA with fully automated apps suggested conceptualisation of the DTA differs from the TA. Tong and colleagues (2022) proposed that the role of bond, as conceptualised in the TA literature, may be less applicable in the DTA context, and that human emotions such as empathy are hard to achieve in digital contexts. The studies reviewed assessed the DTA using quantitative scales that were originally designed to measure the more traditional TA,^26,32,33^ such as the Working Alliance Inventory-Short Revised (WAI-SR^34^) and the mobile Agnew Relationship Measure (mARM).^35^ Both measures are based on Bordin's (1979) alliance model, but substitute items for the digital context; for instance, the mARM replaces the word ‘therapist’ for ‘app’. Using such methods to develop measures assumes that the dimensions of the DTA are similar to those of the TA; however, further empirical investigation is needed given the DTA seems to have a weaker association with therapy outcomes than the TA,^27^ and that human concepts may not be transferable to the digital context.^31^

A recent qualitative study by Tong and colleagues^36^ with 20 users of mental health apps explored the key dimensions of the DTA with fully automated apps. Findings from this study showed that the DTA can be built through accountability, openness, flexible interactions, emotional support and connections with apps. Accountability refers to the individual's willingness to start and maintain the relationship with the app and openness refers to the degree to which individuals feel able to report on personal information. Flexibility was defined in terms of time, location, duration and function. Although the authors argued that the concept of bond was less important in the digital context, individuals did feel a sense of emotional connection and support from the apps. Additionally, the role of goals, a core component of Bordin's conceptualisation of the TA, was not found to be a key process within the DTA. This finding contradicted a study by Darcy et al.,^26^ which found that goals were rated highest on the WAI-SR, with the ‘Woebot’ chatbot app, a finding also supported by Prochaska and colleagues’.^37^ A limitation to Tong et al.'s^35^ study was the use of a non-clinical population, particularly as research suggests non-clinical populations have a more positive attitude towards DHIs.^38^ A further limitation is that the sample was not culturally diverse, which is a particularly important limitation given research suggests that marginalised groups are more likely to access mental health support through DHIs due to ease of accessibility, issues of stigma and financial costs.^39,40^ More research is therefore needed to understand the role of the DTA across a heterogeneous population, including people with mental health problems and across diverse cultural backgrounds. Given the inconsistent findings in the literature, further exploration and clarity around the DTA in relation to the role of goals is also required.

To address these gaps in the literature around the nature of the DTA in the context of standalone apps, and continue to advance our conceptualisation and understanding of the DTA specifically from a diverse group of users experiencing mental health difficulties, our aims were to explore: (1) whether people form an alliance with a DHI delivered by standalone apps; and (2) what constitutes the DTA with standalone apps (i.e. without human support). Qualitative methodology was used to allow for in-depth exploration of user's experience of the DTA, which is a still relatively nascently explored area. As research to date has largely concentrated on quantitative methods of DTA measurement, it is important to explore the construct subjectively to explore whether additional features not captured in current DTA measures might be important.

## Methods

### Design

Qualitative design with online, individual semi-structured interviews with individuals who were currently using, or had recently used, existing apps downloaded from app stores, for mental health support. Data was analysed using Braun and Clarke's^41^ reflexive approach to thematic analysis. A critical realist epistemological and ontological stance was adopted,^42^ which recognises that data cannot directly represent reality but is rather influenced by one's experience and position within the social world.^43^ Consequently, the researchers considered their reflexive positioning throughout the study process.

### Participants

Eligibility criteria: (i) individuals who self-identified as having a mental health difficulty, and either currently, or in the past six months, were receiving mental health care treatment and/or had a mental health diagnosis; (ii) currently using/used a smartphone app specifically for mental health difficulties (within the last six months, in order to be able to accurately recall and reflect on their experiences), (iii) English speaking (due to not having translation costs); (iv) aged 18 or over; and (v) capacity to provide informed consent. The criterion for using a smartphone app for mental health difficulties was guided by the National Institute of Mental Health 2020 classification of apps, which included apps that facilitate self-management of symptoms/illness, cognition improvement, skills-training, social support, and assessment. Users registered their interest with author RB who screened potential participants against the eligibility criteria. One user withdrew following consent due to personal circumstances. A final sample of 17 users took part. Participants were offered a £10 voucher to reimburse their time and contribution.

### Procedure

Ethical approval was granted by the University Research Ethics Committee (UREC reference 2020-10206-16772). Participants were recruited via social media and through a university counselling service and University email bulletins and posters. Attempts were made to recruit a sample that reflected people who had experienced a range of mental health difficulties, across a range of backgrounds and ages, in line with qualitative methodology of exploration of different experiences.^44^ Therefore, a log was kept monitoring individual characteristics and where gaps were identified a more purposive approach to recruitment was used to target certain populations. For example, we initially recruited a higher proportion of female participants so adapted our advertising materials to highlight that we were particularly interested to hear from males. Participants provided written informed consent and completed a series of questions collecting demographic information and questions around app use to contextualise the sample. These questions were piloted with a lived experience advisory panel. Interviews were conducted by author RB, who had training on qualitative interviews during clinical psychology doctoral training, and training with feedback on interview technique and style with qualitative researchers offering extensive qualitative experience whilst working on a large-scale research project. RB also had prior experience conducting qualitative interviews. Interviews followed a topic guide (Supplemental Table 1) developed by the research team and informed by previous literature and Bordin's (1979) TA conceptualisation model. Input on the topic guide, specifically, the content and language, was received from a lived experience advisory panel; the topic guide was adapted based on feedback. Pilot interviews were also conducted to help the development. Interviews took both an inductive and deductive approach (drawing on the in-person alliance). Initial interview questions explored general experience of apps. Open-ended questions pertaining to TA experiences were in response to users spontaneously reflecting on an alliance or using alliance terminology (e.g. connection). However, if, towards the middle of the interview, the user had not described any experiences in relation to an alliance, specific questions were asked. For example, the interviewer explored views on the idea of a connection, and goals were specifically explored to reflect the lack of clarity around this latter concept identified in the literature.^36^ The topic guide was revised to allow for additional exploration of areas that surfaced in the initial five interviews. Interviews took place via online videoconferencing platforms were audio-recorded using an encrypted digital recorder and lasted between 35 and 75 min (M = 47 min, SD = 9.4). In line with qualitative research, recruitment ended when data sufficiency^45^ was reached; specifically, based on analysis of transcripts and an agreement within the research term that no additional themes were generated from the data.

### Data analysis

Interviews were anonymised and transcribed verbatim by author RB. Transcripts were stored and managed in NVivo.^46^ Pen and paper methods were also used to support final theme generation for a deeper immersion into the data. Thematic analysis^39^ was considered the most appropriate method to analyse the data because of the flexibility it offered to structure and organise the data, allowing for both inductive and deductive theme generation.^47^ Specifically, some codes were informed by existing theoretical constructs around the TA (i.e. goals), thus resembling a deductive approach, alongside researchers seeking patterns in the data to build theory around the DTA, offering an inductive approach.^48^ Data analysis consisted of the following six stages:^39^ (i) data familiarisation (accuracy checking of transcripts and familiarisation with dataset by repeated reading of transcripts and listening to audio recordings, facilitating immersion); (ii) initial code generation (author RB organised the data by semantic coding on NVivo; authors SB and KB independently coded two transcripts to ensure rigour); (iii) generating (initial) themes (codes grouped together and mapped onto provisional themes to promote flexibility with generating themes); (iv) reviewing themes (data set was reviewed by the authors to refine themes, ensure themes were reflective of the source data and related to the aims of the research); (v) theme defining and naming (themes finalised by cycling between the data and themes to organise the story in line with the research question; quotes identified to support themes); and (vi) report production. Throughout coding and theme generation, differences in opinions between the authors were managed through reflexive discussions and reviewing the data together. There was no attempt to determine intercoder reliability, as this can contradict the interpretive agenda of qualitative research.^39^

### Reflexivity and rigour

Author RB kept field notes and a reflexive log following interviews.^49^ This process of reflexivity was used to help ensure rigour and quality of the research through explicitly stating and understanding how the researchers’ position and experience impacted the data.^50^ For example, reflective logs were used to consider how interviewer questions and interviewee responses influenced researchers’ own views about the topic. Author RB is a trainee clinical psychologist, new to the field of the DTA, with clinical therapeutic experience working mostly in-person with individuals. Authors SB and KB have experience in digital mental health, are involved in clinical trials implementing DHIs, and are experienced qualitative researchers. All authors acknowledge that their experiences may have contributed to the collection and interpretation of data, including interpreting data in terms of relationships. Notably, the authors were aware of intersecting relationships between themselves and the research users and their identity as a healthcare professionals and researchers.

## Results

### User characteristics

Seventeen (3 male, 14 female) participants aged 21–52 years (M = 30, SD = 8.3) took part. All participants self-identified as having a mental health difficulty, with the majority having received a formal diagnosis (13/17, 76%), including depression, anxiety, personality disorders, schizophrenia, bipolar, post-traumatic stress disorder, and obsessive-compulsive disorder. Some participants were currently in receipt of mental health services (6/17, 35%), and most had previously, or were currently accessing, psychological therapy (9/17, 29%). Around half of participants were from black and ethnic minority backgrounds (8/17, 47%). Participants characteristics are summarised in Table 1. All participants were currently using apps for mental health support. Table 2 describes app characteristics; apps used by participants included mindfulness and meditation (*n* = 19), tracking apps (*n* = 7), therapeutic skills (*n* = 7), chatbot (*n* = 2), psychoeducation (*n* = 2) and journaling (*n* = 1).

**Table 1.** Participant demographics.

Demographic information	*N*	Percentage
Age		
18–24	6	35.3%
25–29	2	11.8%
30–34	5	29.4%
35–39	3	17.6%
40–44	0	0.0%
45+	1	5.9%
Gender		
Male	3	17.6%
Female	14	82.4%
Ethnicity		
White British	9	52.9%
British Asian	5	29.4%
Mixed/multiple	3	17.6%
Employment status		
Employed	6	35.3%
Self-employed	1	5.9%
Student	6	35.3%
Unemployed	4	23.5%
Living situation		
Lives alone	5	29.4%
Lives with partner	3	17.4%
Lives with others	9	52.9%
Marital status		
Single	12	70.6%
Co-habiting	1	5.9%
Married	2	11.8%
Divorced	2	11.8%
Mental health diagnosis?		
Yes	13	76.5%
No	4	23.5%
Current mental health service involvement?		
Yes	6	35.3%
No	12	70.6%
Currently receiving psychotherapy?		
Yes	4	23.5%
No	13	76.5%
Previously received psychotherapy?		
Yes	5	29.4%
No	12	70.6%

**Table 2.** Details of apps participants used.

Participant	Goals for using mental health app support	App/s used	Main function of app/s	Time using apps	Frequency of app use
P01	To better manage mood and to feel calmer	Headspace	Meditation	2 years	Daily
P02	To ground self when dissociating	Brain in hand Insight timer	Traffic light system to manage mental health and access support Meditation	2 years	Monthly
P04	To help with sleep and anxiety	Headspace	Meditation	1 year	Weekly
P05	To help with sleep and anxiety. To feel calmer	Headspace Calm	Meditation Meditation and sleep	8–9 months	Weekly
P06	To support overcoming challenges with anxiety, i.e. appointment attendance	Headspace Brain in hand	Meditation Traffic light system to manage mental health and access support	4 years	Daily
P07	To reduce daily anxiety and stress symptoms by calming self	Balance My life Headspace	Meditation Meditation Meditation	2 years	Every few days
P08	Document thoughts to identify patterns and mood fluctuations	Pixel	Journaling and mood tracking	3 months	Daily
P09	General support for wellbeing- to improve focus and sleep	Headspace Calm	Meditation Meditation and sleep	3 months	Daily
P10	To help relax before bed and to keep on track of positive activities	Insight timer Habit tracking	Meditation Behaviour tracking	18 months	Daily
P11	To reduce self-harm following the recommendation by mental health services	Clear fear Calm harm Cove I am sober	Anxiety management Manage urges to self-harm Music therapy Habit tracking and motivation	Several years	Monthly
P12	To help with anxiety by getting thoughts down and to relax. To track mood fluctuations and patterns	Pixel Wysa	Journaling and mood tracking Chatbot	3–4 months	Daily
P13	To reduce anxiety and improve sleep	Headspace Calm	Meditation Meditation	5 years	Weekly
P14	To try to feel better and improve sleep. Could not access therapy so tried apps	Headspace Calm Calm harm	Meditation Meditation Manages urges to self-harm	1 year	Daily
P15	To reduce self-harm and to help build an emotional language to name feelings	Tomo Calm harm Today I am	Mindfulness Manages urges to self-harm Increase emotional literacy	1 year	Every few days
P16	To stay calm and be able to clear mind and distract self when feeling low or anxious	Smiling mind Abide Calm harm Health assured	Meditation Meditation Manages urges to self-harm To track health	2 years	Daily
P17	To develop strategies to apply when alone	Self-help for anxiety management Headspace For me	Anxiety management skills Mindfulness Self-help information	5 years	Weekly
P18	To manage anxiety symptoms	Wysa	Chatbot	1 year	Monthly

### Contextualising app usage

Participants described initially seeking support from apps for several reasons, including: (i) support being unavailable from services and subsequently apps being recommended by professionals (‘…*I was told* w*e [a mental health service] have a waiting list, here's some apps instead’*, Participant 11); (ii) reservations around confiding in people (‘*I think I just kind of struggle to communicate kind of generally with other people anyway, so I think the app is, just a bit, it's a bit easier’,* Participant 08); and (iii) in response to marketing strategies promoting apps (‘…*headspace is advertised a lot on, erm TV. So, I saw about headspace, and I also read it in erm online, in a newspaper as well, so it was promoted, so I downloaded it’,* Participant 05). Furthermore, users described using apps for: (i) mood tracking (‘*You can track it, so you can look back on diaries tracking mood’,* Participant 15); (ii) symptom management (‘*helping you to calm down in that moment’,* Participant 13); (iii) messenger functions (‘…*the one with chatbot’*, Participant 18); and (iv) therapeutic skills *(‘I use them mainly for meditation and mindfulness’*, Participant 05).

### Key themes

**Figure 1.**
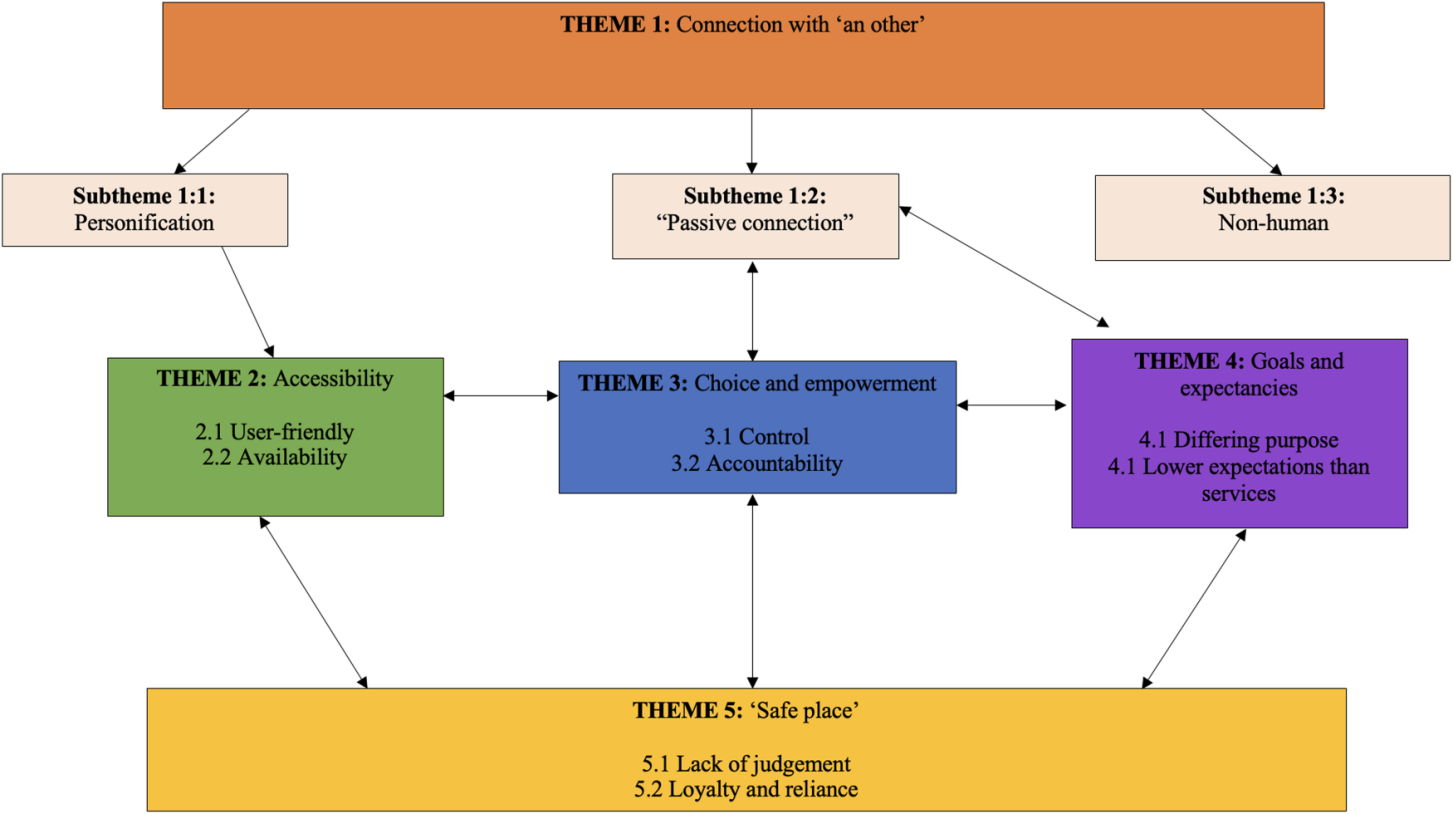
Thematic map.

Five distinct but inter-related themes were developed: (1) connection with ‘an other’; (2) accessibility; (3) choice and empowerment; (4) goals and expectations; and (5) ‘safe place’. There were 11 subthemes across the themes, summarised in Table 3. Theme one pertains to whether an alliance can be formed with an app, and themes two to five relate to the key components for conceptualising the DTA, drawing on comparisons from in-person alliance. The theme names were selected to represent the core concept of the theme. With the exception of the goals, themes did not relate directly to the traditional TA subcomponents. However, there was overlap between the accessibility theme and the previously identified flexibility subcomponent of DTA and the safe place theme and the previously identified openness and emotional connection subcomponents of DTA. The inter-relationships between these themes and subthemes are presented in Figure 1. Relationships between themes were determined through discussion amongst the authors. We discussed different possible inter-relationships between themes based on our knowledge of the data and existing theoretical models of alliance. The final figure represented the most parsimonious representation of relationships. See Supplemental Table 2 for further quotes pertaining to the themes.

**Table 3.** Overview of themes and subthemes.

Themes	Subthemes
1. Connection with ‘an other’	1.1 Personification 1.2 ‘Passive connection’ 1.3 Non-human
2. Accessibility	2.1: User-friendly 2.2: Availability
3. Choice and empowerment	3.1: Control 3.2: Accountability
4. Goals and expectations	4.1: Differing purpose 4.2: Lower expectations than services
5. ‘Safe place’	5.1: Lack of judgement 5.2: Loyalty and reliance

### Theme 1: connection with ‘an other’

There was a consensus that apps foster some form of connection with participants. However, there was variability in terms of how individuals perceived and conceptualised this connection. The theme of connection with ‘an other’ was underpinned by three subthemes: (1) personification; (2) passive connection; and (3) non-human.

#### Subtheme 1.1: personification

A small number of participants personified the app they used for their mental health, describing the association with the app as akin to a human relationship, representative of a friendship. For example, users felt that the app/s they used offered commitment and loyalty simply through knowing the app was always available (theme 2). This was commonly cited with those who had less access to human support, such as in cases where people were living alone:

*I’ve considered the app to be friend of mine, like it's there, and I know it's there to help me…sometimes it is saying that, you know, technology is more loyal than friendships…That's why I consider the app to be a friend, it can be a resource where I can turn to, there's a sense of commitment.* (Participant 05)

#### Subtheme 1.2: ‘Passive connection’

Others described a ‘passive connection’ with the mental health app they were. These users felt connected to the app content or what the app facilities (i.e. a connection with others) as opposed to the app itself and were clear that this connection was distinguishable from a interactional human relationship. Apps were felt to have the ability to emulate some human therapist traits and qualities through the ability to ‘check in’ and offer a space to describe feelings. This ‘offload’ feature provided a sense of being heard and held (‘*It kind of feels like it will hold onto it for you’,* Participant 04) and was cathartic in the same way that being heard by another human being can be:

*Sometimes it feels almost like you have gone into a little room, like a cosy room and just someone sitting there, and you've just sat down talked to them for a bit they just sort of nodded at you [laughs]. And then you've left it, it feels, it does sometimes feel like you've left something behind in there, you know.* (Participant 09)

Apps that included a human voice facilitating exercises were more likely to generate a sense of connection with the app for participants. Although participants could not see the person, or have a two-way interaction per se, the feature of a voice facilitated a sense of feeling understood and connected to the person speaking, thus fostering alliance with ‘an other’:

*… I mean, at the end of the day with [app name] it's just ‘Andy's voice’, but I feel like he understands. I don't know, it's kind of weird. I feel like there's somebody out there, at least, I feel like things that are designed so well to involve some sort of understanding of the person on the receiving end and that I feel connected with that…* (Participant 01)

Others described apps as facilitating wider connections such as fostering a sense of connection to others using the app (‘…*it tells you how many people are meditating with you at the same time, so you don’t feel alone’*, Participant 04) or enabling better relationships with services. Relatedly, DHIs were sometimes viewed as an extension of the clinical team, which created a connection with mental healthcare workers outside of mental health appointments:

*…a psychiatrist knows you're not going to tell him everything [laughs]. He knows my patients are going to be a little bit loose… So this is saying* [referring to the app], *‘Look, I've done all these things, this is where I'm at, and this is the evidence,’ and he's saying, ‘yeah, well done, you’re going in the right direction’. So it's not about him. It's more about me but also, it's about relationships, building a relationship with him.* (Participant 06)

#### Subtheme 1.3: non-human

Other participants described apps as disingenuous (‘…*but, of course it wouldn’t be genuine’*, Participant 12), lacking empathy, and the inability to offer the nuances in interactions that create relationships. Most users who were currently receiving therapist support identified feeling apps could not replicate human qualities in the same way a human could, describing apps as emotionless and concepts such as bond and relationships with apps as untenable. In some instances, users likened apps to a robot or held in mind that the AI aspect of app, leading to their experiences feeling invalidated:

*Like as good as computers and AI technology gets, it's never going to be another human, and it will never understand…it is not going to take into account that I am human and the things I’ve lived through very much influence what I experience now … you may use the app and it's just like, it's throwing it in your face, like, ‘Hi, I’m a thing, I have no emotion, I do not feel, I am incapable of feeling, and, here's what you should do because I can just do it. It's like a robot…* (Participant 11)

### Theme 2: accessibility

#### Subtheme 2.1: user-friendly

All participants talked about the ease of using apps and the ability to receive support effortlessly, anytime, anywhere. Participants identified that this factor was important in influencing their engagement with the app:

*…it's got so much choice and it breaks it down in so many ways, so flexible and you can fit it in anywhere in anyway in your life so and it's so easy to use.* (Participant 16)

The function within some apps of selecting unique character animations and profiles helped personalise the app. The familiarity of the profile facilitated a connection, with some users comparing the animation to a friend:

*… they both use really cute like animations on their, erm, like interface so it's kind of like a little, like, it's really recognizable so it kind of feels like erm, like a little friend.* (Participant 09)

#### Subtheme 2.2: availability

All participants valued the fact that apps offered support on demand (‘*…when I'm struggling, I can press that button’,* Participant 16). The convenience of apps was particularly important for those with limited support or through the night-time, which most users identified was a time when they most needed support for their mental health:

*I live alone so I don't have access to other people… and usually I feel at my lowest during the night. And at that time, pretty much everybody is sleeping so I don't have that access. So then, I just basically have the apps.* (Participant 01).

All participants noted that apps were much more accessible compared to clinical services where multiple barriers to access are met, including long waiting lists, even for crisis support (‘*You can access it [apps] whenever you want, and I was on a waiting list to receive therapy for about a year and a half after I’d tried to commit suicide’,* Participant 14). The availability of apps contributed to initial engagement and over time a sense that apps were loyal and trustworthy (theme 5) and available if needed:

…*it's like a safety net, because obviously [laughs] your phone is always on you, so you always know in the back of your head that there is help available.* (Participant 09).

### Theme 3: choice and empowerment

#### Subtheme 3.1: control

Most participants perceived that apps enabled increased control over their mental health care through maximising choice about a range of different issues such as whether to engage with an app in the first place; which app to choose; when they engage; and how they engage (‘*…you can choose to use it how you want basically’,* Participant 06). The increased levels of choice and control that apps provided diminished power imbalances that participants described they experienced when accessing services. Specifically, users described being recipients of treatment with services, through the lack of involvement in decisions around their care:

*…so therapy I have no choice, it's once a week, that particular time, 45 min with a particular lady who only offers one thing, and that will be coming to an end whenever my set of free sessions run out…* .(Participant 14)

Choice and having control were particularly important for people who described their mental health as unpredictable and lacking control over their feelings. In this respect, apps contributed to an improvement in mood and self-esteem:

*If I know I'm doing it* [using an app] *I feel good inside, it kind of helps a little bit with my self-esteem and you know, this is one thing in my life that I can control. See with all the uncertainty with my depression because I can't control when it comes on, I can't control when I'm feeling like that. So, this genuinely feels like something that I can control and that kind of helps in a way.* (Participant 01)

#### Subtheme 3.2: accountability

Participants described how app use made them more accountable for their mental health compared to experiences in face-to-face therapy. App use required self-motivation because there was no-one else encouraging them to engage (theme 1). Having to hold themselves accountable for making decisions around when and how to use app support influenced a sense of ownership over managing their difficulties and treatment. Therefore, participants had lower expectations with apps compared to expectations they had in relation to the support they should receive from mental health services (theme 4):

*… ultimately you have to make that choice to use the app, you have to be in a place where you can be like I want to self-harm, so I’m going to open up the app. Then once you’re on the app you have to be like, ok I’ve got this option, I’ve got this option, which am I going to choose? It can’t make you do anything, it physically cannot and it's not gonna, so, essentially, the app doesn’t do anything, you’re making all the decision.* (Participant 11)

When participants felt motivated to use the app and improve their mental health, this was balanced with good availability of the app (theme 2), leading to a sense of reciprocity within the app-user dyad:

*So, I am committed, and the app provides me that level of commitment at the same time.* (Participant 05)

### Theme 4: goals and expectancies

#### Subtheme 4.1: differing purpose

All participants identified having initial goals when using apps for their mental health. These goals included: symptom management (‘*get me to sleep better’,* Participant 09); mood tracking, (‘*track my mood’,* Participant 08); self-development (‘*goals of understanding myself, building resilience and strength’,* Participant 01); and having access to a form of support (‘*…the first goal was kind of having that safety net’,* Participant 13). Most participants identified with the goal of accessing support and it is this goal that leads to a sense of apps as reliable and trustworthy (theme 5); an important characteristic for the DTA. Goals were viewed differently for in-person therapy compared to app use (‘*… my aim for using apps is very different from my aim of talking to people’,* Participant 01). Overall, goals for app support were seen as short-term symptom-focused, rather than recovery-focused. Apps were seen as having an inability to tackle root causes (‘*…it doesn’t get to the root of everything that's going on’*, Participant 17), or work with trauma. Seeing apps as offering quick solutions for symptoms, as opposed to tackling longer-term difficulties, meant expectancies around app support differed:

*I knew it [apps] would help me in the shorter-term, in terms of dealing with the immediate stress and worry. I think that was better than nothing at that point, but I don't see any sort of app as long-term, especially if you're going through something really traumatic.* (Participant 07)

#### Subtheme 4.2 lower expectations than services

Participants reflected on having higher expectations when accessing in-person support in comparison to support from an app (‘…*the pressure is a lot more on the human’,* Participant 7). They often had little expectations about whether or not the app would help them (‘…*just see whether it works, like there's no harm downloading it’,* Participant 17). In this respect, apps were used as an outlet to share rather than ‘fixer of problems’, supporting the idea that participants themselves took ownership of the problems when using apps (theme 3) and the notion that apps provided a safe space to share feelings (themes 1 and 5):

*…when I’m using the app, like I’m not really expecting much from them, but I know that like I can say whatever I want, ‘cos, I know it's an app and they’re not gonna be able to give me a response but it's like, when you’re using that app, it's less about their response, it's more about just having an outlet.* (Participant 12)

Once individuals felt the app was helping, this led to continued engagement and strengthened alliance building:

*… I just wanted to see what it was about and see if it could help me. So, the breath work I found really useful. And then, once I sort of stayed consistent with it and found that it was helping me, I wanted to stay even more consistent.* (Participant 07)

### Theme 5: safe place

#### Subtheme 5.1: lack of judgement

All participants perceived that apps provided a non-judgemental space, which allowed them to work through difficulties without feelings of guilt, shame and stigma. Relatedly, apps were reported as being more a socially acceptable form of support than visiting a mental health professional than (‘*I think it's much less stigma around apps, than like going to an appointment’,* Participant 17), increasing engagement and connection to apps. One male user from an ethnically minoritised background shared a preference for apps due to mental health stigma:

*…when I tell people I’ve got a mental health problem, people get a bit you know there a bit like, it's a bit like death isn't it- they don't always know what to say … it's sometimes an awkward conversation and as a bloke, but also someone from a BME background it's still a taboo you know…these apps allow me to manage my day-to-day life.* (Participant 06).

Participants reported that sharing personal information with apps was their choice and therefore felt that apps placed fewer demands on them than other types of support. Apps also offered a safe platform, whereby participants felt able to be more open and honest sharing information with reduced concerns around confidentiality, compared with confiding in a person (‘*… like in conventional therapy where they’ll sort of write to your doctor, they’ll write to any therapist that you’re involved with, they’ll write to my mum’,* Participant 15). One participant likened their experience with apps as having a ‘secret diary’:

*Knowing that it's private, it's not uploaded anywhere, no one can access it, it's kind of like having a secret diary, with like a little padlock on it.* (Participant 08)

Participants described expressing themselves more freely with apps, with fewer worries around sharing negative feelings or past trauma with an app, compared to sharing feelings with a person. Notably, common concerns when making disclosures to people were around evoking negative emotions in the recipient, worry about the capability of others managing the disclosures, and being a burden. These worries were not applicable when sharing with apps:

*…one of the things that I often worry about when I am having talking therapy is what I say and how that might affect the therapist. I have been through some quite bad stuff, erm, so I sometimes worry about them. I don’t have to worry with an app because it isn’t a person and I can’t hurt its feelings. If I have an emotional reaction to something that we are talking about, erm, with a person then I might not feel able to be completely, to let that emotion out fully. Whereas, I can’t, I can’t make an app upset. Whatever I do will not elicit an emotional response in an app, therefore it might be possible to be free-er with what, what I say or what I express.* (Participant 02)

#### Subtheme 5.2: loyalty and reliance

There was the overall view that apps offer greater predictability than people through consistent and predictable support, leading to increased trust (‘*… you can reach out when you need it, and it will be like always there for you’,* Participant 09). Relatedly, the ending of app support was also on the participant's terms, unlike with people and services. For most participants, apps were viewed as being more loyal than people (*…‘an app is not going to say, yes, we will call you back within the next hour, and then they don’t call back for five, six, seven hours, or don’t call back at all’,* Participant 02, when reflecting on an experience with clinical services). The predictability and authenticity of apps by not making ‘false promises’ created a safe place, another important factor for alliance building:

*…you just do not know what to expect from people, whereas if you are relying on an app, you know that there's an engagement, there's a commitment, where you are continuing, so you feel more connected and you feel more engaged in the long term* (Participant 05)

## Discussion

### Summary of findings

This study is the first qualitative investigation of the DTA with people who reported experiencing mental health difficulties. Five distinct but inter-related themes were developed from the data: (1) connection with ‘an other’; (2) accessibility (24/7 availability and ease of use offered by apps); (3) choice and empowerment (participants appreciate having control over how they use the app, enabling them to tailor their experience according to their needs, fostering feelings of empowerment); (4) goals and expectations (participants hold themselves more accountable for achieving goals with apps and have different expectations compared to in-person support); and (5) ‘safe place’ (apps provide a non-judgemental, reliable and trustworthy space where users are comfortable to share their difficulties). The findings of this study both extend the current research around the DTA with fully automated app support and provide novel insights into the DTA. Whilst participants described forming a connection with apps, the nature of the DTA with fully automated apps appears nuanced and can be conceptualised differently to the more traditional in-person TA.

The present findings indicate that the dimensions of the DTA cannot be fully captured by adapting Bordin's^21^ three-component model to a digital context. A novel insight into the DTA provided by this study is the alliance as a representation of ‘an other’ through emotional holding and containment. In this respect, apps may serve a similar function to the ‘holding environment’ described by Winnicott,^51^ which, although originally representing the physical and emotional space a mother creates for her baby, has been extrapolated to therapists developing a symbolic holding environment for their patient. Winnicott^48^ argued the primary purpose of a therapist is to provide a holding environment for the patient, which facilitates the person's transition to autonomy. Our findings suggest that apps have the potential to offer a holding environment by allowing people to share their emotions with the DHI, providing a sense of distance and defusion, in turn, making their difficulties feel more manageable. The concept of containment, which involves staying with difficult feelings without adopting a reactive stance,^52^ also has relevance here as users can project feelings onto an app without consequence. This understanding provides valuable insights into addressing common user engagement issues with apps,^50^ whereby apps should prioritise features that foster containment; for instance, through validation, consistent voice features and personalised notifications to enhance engagement.

The notion of containment further resonates with the study findings regarding participants’ goals and expectancies with apps. When using app support, participants do not anticipate receiving responses to their expressed needs. Instead, they accept app support as a safe space for offloading feelings that might otherwise be overwhelming. Thus, individuals establish an alliance by having realistic and attainable short-term goals of app support from the outset. Goals could be achieved collaboratively with an app through process-orientated features, such as mood or habit tracking functions with tailored sequential functions depending on user input and choice. Our finding around the importance of goals reflects Prochaska et al.'s^37^ study exploring Woebot, a fully automated conversational agent, who found goals were rated higher for the DTA than other components on existing alliance measures. Whilst this study has highlighted unique dimensions of the DTA, our finding conceptualising goals as a core component of the DTA is supportive of a core component of the TA model conceptualised by Bordin.^21^

Consistent with previous research,^32,36,50^ the accessibility of apps was found to be a crucial factor in engagement, and therefore a unique dimension of the DTA. Apps being easy to use and available on demand seemed to be a core feature fostering alliance development. A point of difference from previous studies, however, is the finding that apps facilitate a high degree of user choice and control, an important factor for promoting alliance. The perceived power participants identified with when using an app for mental health supported them taking ownership over their mental health difficulties; participants expressed that it was ultimately their choice whether or not to engage with app support. Choice has been demonstrated to increase user engagement.^51^ Users are more likely to hold themselves accountable for their engagement when they have the freedom to decide, when, where, what and how long they engage with content. The increased accountability individuals need to take when using apps compared to in face-to-face therapy might suggest that app support would be most helpful for when they are in the ‘action’ phase of the stages of change model.^33^

The finding that apps offer a ‘safe place’ characterised by non-judgement, loyalty, trust and reduced stigma has been supported by previous research where individuals expressed a freedom to self-disclose, with less apprehension about being judged.^26,32,35^ The absence of fear regarding the emotional impact of sharing information or being judged strengthened the alliance. Furthermore, app support facilitated faster alliance building as it eliminated the need for initial rapport development. The app's ability to create a safe environment with reduced concerns about others is a crucial factor in users seeking refuge in apps during distress, which fosters mutual trust and establishing a DTA. Whilst users value the ability to communicate anonymously, consideration should be given to apps’ ability to address safety during crises, as identified in Koh et al.'s^50^ review.

### Clinical implications and future directions

Considering these findings, apps should be designed in line with the constructs identified to enable the psychological processes that might contribute to lower attrition rates and promote therapeutic change. Findings from our qualitative study suggest that important points to consider when designing apps include incorporating human voice features, apps need to be easy-to-use with customisable profiles, offering choice, goal setting functions, and interventions should be tailored based on individual input. It is also important to highlight that apps may be particularly helpful for certain populations, such as those who do not have access to reliable in-person support. As the DTA remains an emerging field, further qualitative and quantitative studies are needed to either replicate these and/or previous findings, and to further expand the DTA construct. Researchers have begun to measure DTA quantitatively by adapting traditional TA scales and, more recently, by developing and validating a bespoke DTA scale for fully automated mental health apps, grounded in the user experience (Tong et al., in preparation). These efforts will help further elucidate the DTA construct and its relevance and application with digital health engagement and outcomes.

### Strengths, limitations and future research

The study findings significantly contribute to the dearth of qualitative literature exploring the DTA with DHIs. This study has captured the views of people who have directly used apps for their mental health. The inclusion of numerous mental health apps allowed for an in-depth exploration of a range of views of the DTA across a range of apps. A varied sample, particularly in terms of mental health difficulty and ethnic background is a strength, especially because minoritised groups are more likely to access DHIs.^40^ Therefore, our sample likely reflects the views of people who will use and engage with mental health apps. Trustworthiness of the research was achieved through prolonged engagement with data, reflexive discussions between the research team throughout code generation, and revisiting themes and subthemes.

There are also some limitations. Whilst the use of multiple apps allowed for a broad exploration of the topic, an alliance formed with an app may differ depending on the nature, interactability and functions of the app. For example, a meditation app as opposed to a chatbot-based app may offer different alliance features specific to their functions, and as such, this was not explored within this study. Additionally, duration and frequency of app use, as well as mental health difficulty, may impact the DTA. Future research should explore how the type of app, duration and frequency of app use, and particular mental health difficulties impact the DTA.

Furthermore, the interviews took a critical realist stance incorporating both inductive and deductive approaches. Although we attempted to explore the concept by allowing the data to emerge throughout interviews, questions directly pertaining to the TA were asked when relational factors did not organically arise; this approach may have influenced the findings by framing data through a relational lens. The purposive nature of sampling and the use of online recruitment for this study also represent a limitation, within which users might have been especially motivated or interested in using DHIs. Additionally, the sample comprised individuals who self-reported experiencing mental health problems. Thus, the experiences reported may not be representative of the wider population who have a mental health diagnosis and are using apps for support. Furthermore, the sample included more females because more females expressed an interest in the study. Whilst there appears a gender bias, data suggests females tend to use apps more generally,^52^ especially well-being apps. Lastly, it is important to consider the study findings within the context of COVID-19, since users may have a more favourable experience of DHIs given people had to rely more heavily on digital tools.

## Conclusions

The present study provides novel insights into conceptualising the DTA with DHIs delivered by apps for mental health. Users of mental health apps appear to form connections with apps they use to support their mental health. Factors that appear to be distinctly related to the DTA, described as a ‘connection with an-other’ through emotional containment, as opposed to the more traditional in-person TA, include: accessibility; choice and empowerment; goals and expectancies; and providing a ‘safe place’. The need for adapting apps accordingly is emphasised, including the use of human voices to facilitate content, user-friendly and personable interfaces, offering choices and control, building goal features, and functions to be tailored based on users’ input. The field must continue to evolve, balancing innovation with evidence-based interventions to better users’ needs.

## Supplemental Material

sj-docx-1-dhj-10.1177_20552076241277712 - Supplemental material for A qualitative study exploring the digital therapeutic alliance with fully automated smartphone apps Supplemental material, sj-docx-1-dhj-10.1177_20552076241277712 for A qualitative study exploring the digital therapeutic alliance with fully automated smartphone apps by Rebecca Brotherdale, Katherine Berry and Sandra Bucci in DIGITAL HEALTH

sj-docx-2-dhj-10.1177_20552076241277712 - Supplemental material for A qualitative study exploring the digital therapeutic alliance with fully automated smartphone apps Supplemental material, sj-docx-2-dhj-10.1177_20552076241277712 for A qualitative study exploring the digital therapeutic alliance with fully automated smartphone apps by Rebecca Brotherdale, Katherine Berry and Sandra Bucci in DIGITAL HEALTH

sj-pdf-3-dhj-10.1177_20552076241277712 - Supplemental material for A qualitative study exploring the digital therapeutic alliance with fully automated smartphone apps Supplemental material, sj-pdf-3-dhj-10.1177_20552076241277712 for A qualitative study exploring the digital therapeutic alliance with fully automated smartphone apps by Rebecca Brotherdale, Katherine Berry and Sandra Bucci in DIGITAL HEALTH

## References

[1] Vigo DV Patel V Becker A , et al. A partnership for transforming mental health globally. Lancet Psychiatry 2019; 6: 350–356. 30704963 10.1016/S2215-0366(18)30434-6

[2] Coronavirus and depression in adults, Great Britain: July to August 2021 [Internet]. [cited 2024 Mar 5]. Coronavirus and depression in adults, Great Britain: July to August 2021. Available from: https://www.ons.gov.uk/peoplepopulationandcommunity/wellbeing/articles/coronavirusanddepressioninadultsgreatbritain/julytoaugust2021

[3] Bucci S Schwannauer M Berry N . The digital revolution and its impact on mental health care. Psychology and psychotherapy: theory. Res Pract 2019; 92: 277–297. 10.1111/papt.12222 30924316

[4] Pernencar C Aguilar P Saboia I , et al. Systematic mapping of digital health apps–A methodological proposal based on the World Health Organization classification of interventions. Digit Health 2022; 8: 20552076221129071. 36569821 10.1177/20552076221129071 PMC9772938

[5] Musiat P Tarrier N . Collateral outcomes in e-mental health: a systematic review of the evidence for added benefits of computerized cognitive behavior therapy interventions for mental health. Psychol Med 2014; 44: 3137–3150. 25065947 10.1017/S0033291714000245

[6] Kovandžić M Chew-Graham C Reeve J , et al. Access to primary mental health care for hard-to-reach groups: from ‘silent suffering’to ‘making it work’. Soc Sci Med 2011; 72: 763–772. 21272968 10.1016/j.socscimed.2010.11.027

[7] Melbye S Kessing LV Bardram JE , et al. Smartphone-based self-monitoring, treatment, and automatically generated data in children, adolescents, and young adults with psychiatric disorders: systematic review. JMIR Ment Health 2020; 7: e17453. 10.2196/17453 PMC7661256 33118950

[8] Mani M Kavanagh DJ Hides L , et al. Review and evaluation of mindfulness-based iPhone apps. JMIR Mhealth Uhealth 2015; 3: e4328. 10.2196/mhealth.4328 PMC4705029 26290327

[9] Bucci S Barrowclough C Ainsworth J , et al. Actissist: proof-of-concept trial of a theory-driven digital intervention for psychosis. Schizophr Bull 2018; 5: sby032–sby032. 10.1093/schbul/sby032 PMC6135229 29566206

[10] Baumel A Torous J Edan S , et al. There is a non-evidence-based app for that: a systematic review and mixed methods analysis of depression-and anxiety-related apps that incorporate unrecognized techniques. J Affect Disord 2020; 273: 410–421. 32560936 10.1016/j.jad.2020.05.011

[11] Kerst A Zielasek J Gaebel W . Smartphone applications for depression: a systematic literature review and a survey of health care professionals’ attitudes towards their use in clinical practice. Eur Arch Psychiatry Clin Neurosci 2020; 270: 139–152. 30607530 10.1007/s00406-018-0974-3

[12] Sucala M Cuijpers P Muench F , et al. Anxiety: there is an app for that. A systematic review of anxiety apps. Depress Anxiety 2017; 34: 518–525. 28504859 10.1002/da.22654

[13] Firth J Torous J Nicholas J , et al. The efficacy of smartphone-based mental health interventions for depressive symptoms: a meta-analysis of randomized controlled trials. World Psychiatry 2017; 16: 287–298. 28941113 10.1002/wps.20472 PMC5608852

[14] Philippe TJ Sikder N Jackson A , et al. Digital health interventions for delivery of mental health care: systematic and comprehensive meta-review. JMIR Ment Health 2022; 9: e35159. 10.2196/35159 PMC9109782 35551058

[15] Lagan S Emerson MR King D , et al. Mental health app evaluation: updating the American Psychiatric Association’s framework through a stakeholder-engaged workshop. Psychiatr Serv 2021; 72: 1095–1098. 33882716 10.1176/appi.ps.202000663

[16] Carlo AD Hosseini Ghomi R Renn BN , et al. By the numbers: ratings and utilization of behavioral health mobile applications. NPJ Digit Med 2019; 2: 54. 31304400 10.1038/s41746-019-0129-6 PMC6572775

[17] Roepke AM Jaffee SR Riffle OM , et al. Randomized controlled trial of SuperBetter, a smartphone-based/internet-based self-help tool to reduce depressive symptoms. Games Health J 2015; 4: 235–246. 26182069 10.1089/g4h.2014.0046

[18] Torous J Wisniewski H Liu G , et al. Mental health mobile phone app usage, concerns, and benefits among psychiatric outpatients: comparative survey study. JMIR Ment Health 2018; 5: e11715. 10.2196/11715 PMC6269625 30446484

[19] Rapp A Cena F . Personal informatics for everyday life: how users without prior self-tracking experience engage with personal data. Int J Hum Comput Stud 2016; 94: 1–17.

[20] Koh J Tng GY Hartanto A . Potential and pitfalls of mobile mental health apps in traditional treatment: an umbrella review. J Pers Med 2022; 12: 1376. 36143161 10.3390/jpm12091376 PMC9505389

[21] Horvath AO Del Re AC Flückiger C , et al. Alliance in individual psychotherapy. Psychotherapy 2011; 48: 9–16. 21401269 10.1037/a0022186

[22] Holdsworth E Bowen E Brown S , et al. Client engagement in psychotherapeutic treatment and associations with client characteristics, therapist characteristics, and treatment factors. Clin Psychol Rev 2014; 34: 428–450. 25000204 10.1016/j.cpr.2014.06.004

[23] Bordin ES . The generalizability of the psychoanalytic concept of the working alliance. Psychother: Theor Res Pract 1979; 16: 252.

[24] Cavanagh K Millings A . (Inter) personal computing: the role of the therapeutic relationship in e-mental health. J Contemp Psychother 2013; 43: 197–206.

[25] Pihlaja S Stenberg JH Joutsenniemi K , et al. Therapeutic alliance in guided internet therapy programs for depression and anxiety disorders–a systematic review. Internet Interv 2018; 11: 1–10. 30135754 10.1016/j.invent.2017.11.005 PMC6084872

[26] Darcy A Daniels J Salinger D , et al. Evidence of human-level bonds established with a digital conversational agent: cross-sectional, retrospective observational study. JMIR Form Res 2021; 5: e27868. 10.2196/27868 PMC8150389 33973854

[27] Tremain H McEnery C Fletcher K , et al. The therapeutic alliance in digital mental health interventions for serious mental illnesses: narrative review. JMIR Ment Health 2020; 7: e17204. 10.2196/17204 PMC7442952 32763881

[28] Berry N Lobban F Bucci S . A qualitative exploration of service user views about using digital health interventions for self-management in severe mental health problems. BMC Psychiatry 2019; 19: 1–13. 30665384 10.1186/s12888-018-1979-1 PMC6341527

[29] Hollis C Sampson S Simons L , et al. Identifying research priorities for digital technology in mental health care: results of the James Lind Alliance Priority Setting Partnership. Lancet Psychiatry 2018; 5: 845–854. 30170964 10.1016/S2215-0366(18)30296-7

[30] Henson P Wisniewski H Hollis C , et al. Digital mental health apps and the therapeutic alliance: initial review. BJPsych Open 2019; 5: e15. 10.1192/bjo.2018.86 PMC6381418 30762511

[31] Tong F Lederman R D’Alfonso S , et al. Digital therapeutic alliance with fully automated mental health smartphone apps: a narrative review. Front Psychiatry 2022; 13: 819623. 35815030 10.3389/fpsyt.2022.819623 PMC9256980

[32] Clarke J Proudfoot J Whitton A , et al. Therapeutic alliance with a fully automated mobile phone and web-based intervention: secondary analysis of a randomized controlled trial. JMIR Ment Health 2016; 3: e4656. 10.2196/mental.4656 PMC4786687 26917096

[33] Prochaska JO DiClemente CC . Stages and processes of self-change of smoking: toward an integrative model of change. J Consult Clin Psychol 1983; 51: 390. 6863699 10.1037//0022-006x.51.3.390

[34] Hatcher RL Gillaspy JA . Development and validation of a revised short version of the Working Alliance Inventory. Psychother Res 2006; 16: 12–25.

[35] Berry K Salter A Morris R , et al. Assessing therapeutic alliance in the context of mHealth interventions for mental health problems: development of the mobile agnew relationship measure (mARM) questionnaire. J Med Internet Res 2018; 20: e90. 10.2196/jmir.8252 PMC5934536 29674307

[36] Tong F Lederman R D’Alfonso S , et al. Conceptualizing the digital therapeutic alliance in the context of fully automated mental health apps: A thematic analysis. Clin Psychol Psychother 2023; 30: 998–1012. 37042076 10.1002/cpp.2851

[37] Prochaska JJ Vogel EA Chieng A , et al. A randomized controlled trial of a therapeutic relational agent for reducing substance misuse during the COVID-19 pandemic. Drug Alcohol Depend 2021; 227: 108986. 34507061 10.1016/j.drugalcdep.2021.108986 PMC8423936

[38] Schröder J Berger T Meyer B , et al. Impact and change of attitudes toward internet interventions within a randomized controlled trial on individuals with depression symptoms. Depress Anxiety 2018; 35: 421–430. 29489038 10.1002/da.22727

[39] Cummings JR Allen L Clennon J , et al. Geographic access to specialty mental health care across high-and low-income US communities. JAMA Psychiatry 2017; 74: 476–484. 28384733 10.1001/jamapsychiatry.2017.0303 PMC5693377

[40] Ralston AL Andrews III AR Hope DA . Fulfilling the promise of mental health technology to reduce public health disparities: review and research agenda. Clin Psychol: Sci Pract 2019; 26: e12277.

[41] Braun V Clarke V . Reflecting on reflexive thematic analysis. Qual Res Sport Exerc Health 2019; 11: 589–597.

[42] Fletcher AJ . Applying critical realism in qualitative research: methodology meets method. Int J Soc Res Methodol 2017; 20: 181–194.

[43] Vincent S O’Mahoney J . Critical realism and qualitative research: An introductory overview. In: The Sage handbook of qualitative business and management research methods. Sage, 2018.

[44] Patton MQ . Qualitative evaluation and research methods. Sage Publications, Inc, 1990.

[45] Braun V Clarke V . To saturate or not to saturate? Questioning data saturation as a useful concept for thematic analysis and sample-size rationales. Qual Res Sport Exerc Health 2021; 13: 201–216.

[46] QSR International Pty Ltd. NVivo qualitative data analysis software (v10). 2012.

[47] Braun V Clarke V . One size fits all? What counts as quality practice in (reflexive) thematic analysis? Qual Res Psychol 2021; 18: 328–352.

[48] Bryman A . Of methods and methodology. Qual Res Organ Manag: Int J 2008; 3: 159–168.

[49] Ortlipp M . Keeping and using reflective journals in the qualitative research process. Qual Rep 2008; 13: 695–705.

[50] Fink AS . The role of the researcher in the qualitative research process. A potential barrier to archiving qualitative data. In 2000.

[51] Winnicott DW . Transitional objects and transitional phenomena. London: Tavistock, 1951.

[52] Casement P . On learning from the patient. Routledge; 2013.

