# Supplementary material for: A qualitative study exploring the digital therapeutic alliance with fully automated smartphone apps
Source: Digit Health. 2024 Dec 15;10:20552076241277712. doi: 10.1177/20552076241277712 (PMC11648025; doi:10.1177/20552076241277712)
Supplement: sj-docx-1-dhj-10.1177_20552076241277712 - Supplemental material for A qualitative study exploring the digital therapeutic alliance with fully automated smartphone apps [file sj-docx-1-dhj-10.1177_20552076241277712.docx]

**Supplementary Table 1.** Topic guide.

| 1. **General use of digital devices**   **Explore participants views of digital devices more generally, and their feelings about them.** | Please can you tell me a little bit about your current use of digital devices…   - What are you using? - What are you using them for? - How often?   How do you generally feel about digital devices?   - PROBE if needed; some people say they enjoy online devices whereas others say they get annoyed with using digital devices…. |
| --- | --- |
| 1. **Apps specifically**   **Explore participants use of smartphone apps more generally** | Please tell me a little bit about your current app use…   - What apps are you using? For what purpose?   PROBE: To connect with others? For your health - physical or mental health?  When did you first start using apps?   - What types of apps? What for - purpose? - How did you find out about the apps? Anyone recommend the apps? Who? - How did you find them initially? - Did they help? If so, what with? |
| 1. **Apps for mental health**   **Explore participants use of smartphone apps for mental health** | Please can you tell me a little bit about your current mental health app use…   - PROBE: Which apps? - Purpose/primary reason - How often? - Most used/least used? Why?   Any specific reasons for app support?   - What first brought them to using mental health apps? - PROBE: Any reasons why chose apps? Recommended- by who (?services)? Consider different support? - Did they choose an app over other support- face-to-face? If so, why?   Initial goals for app support: What were **your goals** when started using them?   - Do you feel like the app helped you to achieve your goals? How? - How much control do you feel you have in choosing how to use the app and gain support? - Goals similar to when seeking other support? - How much control do you feel you have in choosing how to use the app and gain support? |
| 1. **Feelings about apps**   **Broadly speaking what are your experiences of using these apps?** | How do you generally feel about mental health apps?   - PROBE: Helpful/Not helpful? - Access? Easy to use? - Before you started using an app, did you believe it would help you? - Views changed? Feelings now?   What are the main positive and negatives using an app for mental health?   - PROBE: How do the apps help you? - Conversely, how do they not help? Are there times you avoid using them? When? |
| 1. **Introducing the therapeutic alliance** | Do you feel you have any kind of connection to the app?   - Any kind of relationship? Can you describe this? - If yes, is this connection personal in any way? - Or would you use another word for this? - Connection to what?   Do you feel as though you are in a partnership with the app, like you are working with the app towards a shared goal or purpose?   - Can you tell me more about this experience? - What are your thoughts about the partnership? - Can you think of any examples?   Do you feel you can rely on the app?   - In what way? - Similar/different to a person?   Do you feel you can trust the app?   - In what way? - Similar/different to trusting people?   Do you feel the app is flexible enough to meet your needs?  To what extent do you feel comfortable providing personal information to the app?   - Are there certain things you avoided? - Reasons why? |
| **If the alliance does not emerge:**   - The key thing in face-to-face therapy, is a good relationship with the person and therapist. It is believed that this relationship makes the different. What do you think about the idea that you can have a relationship with an app? - **If no,** how does it differ? - **If yes,** what does this relationship look/feel like? What are the similarities and differences compared to face-to-face? - Some people have likened an app to similar as human support, as such a friend, whereas others have completely rejected this idea, where do you sit with this? - If you have had face to face therapy before, can you describe the differences in the ‘connection/relationship’ with the app, compared with a human therapist? - Do you feel an app can offer any relationship qualities? - How does an app compare with speaking to a person? - How does it compare for helping you? - Are there times you have chosen the app, over a person? - Reasons for this… - What is it like, turning to an app for difficulties? What does it feel like? | |
| 1. **Apps and COVID**   **Explore whether app use changed with COVID-19** | During COVID-19, did you notice any changes in your usage of mental health apps?   - If yes, was this during or after? - Can you tell me a bit more about that difference? - PROBE if needed: Were you using apps more or less? Rely on it? |

**Supplementary Table 2.** Additional supporting quotes

| **Themes** | **Subthemes** |  |
| --- | --- | --- |
| 1. **Connection with ‘other’** | - 1. ***Personalisation*** | *“... it felt like I had someone guiding me, like, I had in therapy, it's kind of like a little, like erm, um, like a little friend…, it definitely helps you feel less isolated, it sounds really dumb [laugh] but it's like, it feels like they're reaching out to you, instead of you having to like do a lot of the work by like physically accessing it, um, and so it kind of just makes you feel less alone, really.”* [Participant 09]  *“There's always that feeling of detachment in the end, but in the process, I feel connected like I do with people… it's just really hard to explain.”* [Participant 01]  *“…sometimes it feels like there's someone there because it's so customizable, especially with balance and it knows when you've not been on there and then that can help you feel like you know it's really been catered to you, and someone is there sort of looking at your needs and how you, um, what you need at the time.”* [Participant 07] |
|  | - 1. ***Passive connection*** | *Erm. I think. It kind of feels like, just like, someone to listen really, like very passive connection, very just just come and say what you need and then you can go, and there's no like judgement, or like response* [Participant 05]  *"It just feels better than just speaking to empty air, sometimes it feels like something is there just listening, but you know very passively..."* [Participant 04]  “…*there might be a person guiding me through the meditation, who isn’t there at that point but hundreds of thousands, millions of people all over the world are meditating right now and I am connected to them in a sense.”* [Participant 02]  *“I suppose, using on a day-to-day basis is some kind of relationship, but it's not, It's not the same as social interaction I'd say…So I think I have got a relationship with the app, because I'm using a day to day basis, but I'm using it to facilitate my life to develop social relationships. I think that's, that's the way I see it. So this helps me develop relationships, this helps me develop independent life, so it plays a part, but I'm more interested in that social interaction, rather than the actual app itself so yeah…it’s a means to an end. So, yeah…”* [Participant 06]  *“It feels like having like a massive group of people that are always like there when you need it.*” [Participant 02] |
|  | - 1. ***Non-human*** | *“…but, of course it* [app] *wouldn’t be genuine, so yeah, you could like program an app to say things like, ‘yeah, that sounds really hard’, but of course, like that doesn’t mean anything….”* [Participant 12]  *“When you use the word relationship, I think about, you know, social relationship, personal relationships, erm…other type of things, dating [laughs] and all those things. But I suppose the way I describe it is screen time, you know, everyone uses their phone or laptop or the iPad and we used to that, that’s a familiar thing to us.* …[Participant 06]  *“.., we’re not created to do life alone. We cannot do life alone, like, it is, maybe it’s possible but it’d be damn right, damn right lonely, and we need people, that, that is the crux of it, but even though, yes, apps are created by people and therefore could they sustain a form human interaction, (sigh) it’s, it, it is that, you, it is a, you don’t know what it’s like for me because you’re just, a series, you’re just some, essentially some code, you are giving me pre-programmed responses, even if it was the most developed AI ever, like, it’s still going to be pre-programmed, pre-calculated responses to an extent where it is essentially just code, it’s not a human with emotions and feelings and compassion and empathy and care and love, and, I could keep going, erm, it’s not, it will never feel,”* [Participant 11] |
| **2. Accessibility** | ***2.1 User friendly*** | *“Erm, I think personally it’s easier with the app because I like get anxious, like with people. Yeah, like, so yeah, I wanna be, like sometimes like I get too anxious to like go to a therapist, so having like, just like my phone, being able to pick up my phone and like, erm, access that, instead of having to leave, erm, and go, make an appointment, like talk to all these people, make an appointment, yeah.”*  *“ I think you can, erm, I guess unpack more I think, more quickly almost, erm, so because you're not having to, I guess get comfortable, erm, you can kind of just begin,”* [Participant 08]  *“So it’s just, erm, like the app like asking you, how you are, and you can type in a response, and then it asks you, so it’s just, erm, (P4) erm, yeah, I don’t know, it’s just like, kind of like, it’s just like a space, like a comforting like space, I guess. Erm, yeah, like manage your thoughts or like, you know, I don’t know.”* [Participant 12] |
|  | ***2.2 Availability*** | “…*although I needed help, I actually wouldn't have wanted to go out and see anyone so being able to just use an app was good for me at that time. Like the truth is, everything is at our fingertips with app support.”* [Participant 16]  *“I've had periods where I’ve not been well at all. And throughout that time I found the waiting list to get help can be very long. Even getting on a waiting list to get help can be very difficult and so, finding ways to be able to help yourself has led me to using apps.”* [Participant 16]  *“I live alone so I don't have* *access to, like other people… and usually I feel at my lowest basically during the night. And at that time, pretty much everybody is just like sleeping so I don't have that access. So then, I just basically have the apps.”* [Participant 01]  *“…it definitely feels, a lot quicker as well, erm, my experiences of calling suicide hotlines, erm, I sort of have to wait for a while, on hold, on in a queue, and it just sort of, I, I can’t imagine it’s really the best way to do things (laughs), so it feels a lot quicker by going onto the apps.”* [Participant 15]  *“You don’t have to worry about like waking someone up, it's just right there. It is always accessible,”* [Participant 09]  *“Em I think it definitely fills the gap like I think I like the fact it’s there all the time, em, cause it means that I don’t have to worry about like being over relying on it or not having it just always there em.”* [Participant 07] |
| 1. **Choice and empowerment** | ***2.1: Control*** | *“Whether it's depression, anxiety, bereavement like anything, whether it's you know. A relationship problem like, like they [apps] literally, the list is endless that you can pick support from.”* [Participant 16]  *“… because I can do it without anyone else that means I can be more in control of what I choose to click on, which strategies I use. If something doesn’t help me, I just skip for a bit.”* [Participant 17]  *“And when you make a decision, you feel you can trust yourself more…you feel good and that's empowering …”* [Participant 06]  “*So kind of empowers my beliefs and my thoughts and opinions, that you know I'm not an outcast in this kind of a race, if you call it. So yeah, it feels empowering when I feel like there are people out there who have specifically done or designed this app or designed these platforms for people like me to come and talk. That's, that's empowering.”* [Participant 01]  *“Yeah like sometimes I think it does make it easier having a app because an app doesn’t have its own like agenda or whatever. If something doesn’t work then I don’t have to explain why it doesn’t work and I think I just skip through it and also like I feel less pressure. like When I was learning strategies through CAMHS I think sometimes it’s kind of hard because I was like negotiating my own anxiety em within a room with someone else and also trying to learn like strategies. Like with an app because you are just stay at home em learning different strategies that can be easier em less stressful than someone like there trying to teach you and be anxious at the same time.”* [Participant 17]  *“So using the apps would be, always would be my choice. Obviously with the insight timer it is my choice, it is up to me whether I engage with that.”* [Participant 02] |
|  | ***2.2: Accountability*** | *“ It is obviously just an app, just like pushing yourself to like go on it and do it, and there's no one else to sort of push you to do that.… the ownness is on you to sort of keep it going, there's no one to push you.”* [Participant 07]  *“Whereas, the app I can chose not to engage, so it is not necessarily always a good thing.”* [Participant 02]  *“If an apps telling me to do it, I totally walked into this app on my own, and I was clearly looking for some kind of support, erm, so, if it’s telling me to do something then, erm, it, it definitely feels a lot less to hearing a person tell me to do something.”* [Participant 15]  *“It* [apps] *offers you I guess independence and so you have to have that, (sigh) that ability, that responsibility for yourself.”* [Participant 11]  *“Em I think it’s reassuring for me because it’s like ‘oh I know that it would be there’ em and I can use it and I can like I guess it’s kinda like empowering me as well because I’m doing it myself.”* [Participant 17] |
| 1. **Goals and expectancies** | ***4.1 Differing purpose*** | *“I didn't think it [apps] would be a long-term solution because I know with anxiety and stress obviously if you don't get to the root cause it always is going to come back.”*[Participant 07]  *“So, when I started using them I thought, okay, I mean this is going to make me feel good for the time being, like, I've still got all the stressors around me 24/7. In my life this stress is always going to be here and I'm going to have to live with them and just move on and carry on with life and this is going to be like a relief for a while, but I now feel like because of the positive, umm, reinforcements and like all the…the way I'm choosing to see things I think that makes the app a lot more appealing to use now. Erm..and yeah, so I think, it is changing my perspective of things, I never really thought I'd be somebody who would one day actually feel really good from it. So I think shifting from that to this end has been I think because of the apps.”* [Participant 01]  *“Yeah, I mean, when you’re having like a lot of negative thoughts, so, erm, so like yeah, sometimes like you don’t necessarily like wanna like put them on someone else, you just wanna like get rid of them, so that’s when an app will be helpful, but if you needed a response, or you need advice, that’s when a therapist, that, yeah, that’s why I said if they work, would work kind of hand in hand, yeah.”* [Participant 12]  “…when you're dealing with something specific it's not something that the app can fix or help you with, so it just sort of sometimes…it just helps the symptoms rather than the actual cause.” [Participant 07]  *“Erm, but I think they’re very different, I think, the app for me is more about calming down and having that kind of, back up’s the wrong word, but, like if everything was really going wrong I’ve got that there, it’s always gonna be there, whereas therapy kind of me treats the whole picture and it’s, it’s not for those intense moments in which an app are for me, it’s more to kind of overall treat you and make you feel better in six, seven weeks time, erm, whereas the app for me is sometimes more of an emergency thing, if I really feel like I need it at the time, erm, and yes before it was probably used as more of a consistent tool, but for now it’s more of a, as and when I need it, erm, so I think they’re quite different. For me the therapy is about treatment and about, erm, understanding and you know getting you to a point where you’re starting to feel better, and the app is more about, I just don’t think an app can fully understand your personal issues, so, it might be one really specific element of OCD that’s bothering you that day and I don’t think an app can know to treat that specific thing. I think it’s more for holistically helping you to calm down in that moment.”* [Participant 13] |
|  | ***4.2 Lower expectations than services*** | *“Umm…so like with the app, it can't, it can't talk to you it can't emote with you. So you are, it’s the benefit of the doubt that you have to give it… but with a human it’s like, okay, this person is a living, breathing person and so the pressure is a lot more on the human. Which is why I think if it's disappointing, it feels a little worse than you, that it would with an app.”* [Participant 01]  *“The aim was mainly, do you know just to engage in the programme, so I went in just to engage with the app. Try to learn, to develop, to grow and er supporting me with my own personal development and erm my mental health. So um that’s my initial thoughts of er the app.”* [Participant 05]  *“I didn't realize I needed an app, so you know when I when I joined the mental health services I didn’t realize you know, I would need support and when you need support and no one's there, the apps there for you kind of thing. It's there all the time. So, you know, it's just so nice to have something that can support you and you can do it yourself. So I didn't realize I would need an app or I didn't realize it was so beneficial but after using the app or after using some of the, you know the the content, using some of the features I found out that actually, you know, it's beneficial. Even if I use it once a month it's beneficial.”* [Participant 06]  *“I think to start I just didn’t really have that many goals I think it’s more just or just see whatever it works like there’s no harm downloading it and I think the I guess went on to download it I guess I just wanted to have something there em yeah I think that was kind of my goal just having something that I can use*.” [Participant 17] |
| 1. **Safe place** | ***5.1 Lack of judgement*** | *“Erm, I think, erm, it helps, I think if sometimes when you’re talking to like a person just you might just leave out things, or not be really honest with yourself, ‘cos of that fear of like being judged is always there, but then it’s like if you’re with the app, like I know it’s like just an app and like, erm, it doesn’t like store, erm, I think there’s a setting like, it doesn’t like store the journal entries if you want it to like go away at the end of the day, which is, that’s what I prefer, so I’ll just type, or I just say like whatever, and you know, and I know there’s like, it, yeah, it, erm, it’s like, it’s just a little easier, I guess.”* [Participant 11]  *“Erm and I think using the APP kind of removes, I guess, an element of, I mean obviously that's what therapists and that kind of thing that that’s what they are there for, they are there to work through feelings of like embarrassment, guilt and shame and that kind of thing. Erm, and obviously with an app, like that that human element isn't there so you don't have to worry about judgment, I think.”* [Participant 08]  *“…one of the things that I often worry about when I am having talking therapy is what I say and how the therapist might, not think of me necessarily, but how that might affect the therapist. I have been through some quite bad stuff and erm, so I sometimes worry about them. I wouldn’t have to worry with an app because it isn’t a person and I can’t hurt it’s feelings. If I, if I have an emotional reaction to something that we are talking about, erm, with a person then I might not feel able to be completely, to let that emotion out fully. Whereas, I can’t, I can’t make an app upset. Whatever I do will not elicit an emotional response in an app, therefore it might be possible to be free-er with what, what I say or what I express.”* [Participant 02]  *“I can go on there* [referring to apps], *it’s right there, it’s ready for me, erm, and it doesn’t feel like I’m, gonna be heard by several people, erm like in conventional therapy where they’ll sort of write to your doctor, they’ll write to, erm, any therapist that you’re involved with, they’ll write to my mum, erm, so, I, I’m perfectly aware that it’s like, it’s for safety and like, erm, I guess just for records as well, erm, but, it can almost feel like I’m talking to about five people, erm, when I do conventional therapy, and whereas say Tomo* [app], *I, I kind of, it’s, it’s all anonymous, no one even knows I’m doing it, erm, and I can just go on there and it’s right there available, I’m not gonna get a letter about my experiences or what I was feeling, erm, that day (laughs) so…. Erm, it kind of allows me to be more open I think, erm, with certain therapists, if I don’t trust them, or I don’t feel like they’re very helpful for me, erm, it definitely allows you to be a bit more reserved and you don’t really talk about everything maybe you need to talk about, so, I definitely like I can be a lot more open, ‘cos it’s all anonymous and there won’t be a record of it saying, yeah, erm, (laughs) hello mother of me, you know (laughs) so this is what your daughter was feeling and everything she said, everything, it, it definitely feels a lot more like I can be free and it wont come back to haunt me (laughs) at any point.”* [Participant 15]  *“…sometimes dealing with people is hard, because there is that, embarrassment, shame perhaps, erm, you just want to feel ok and you need, you, you don’t, yes, other people will help a lot, but you don’t want them there at that point, and so, yes, that is where apps can be useful… people don’t need to know that they’re there, people don’t need to know that you’re using it.* [Participant 11]  *“… like a lot of people I know use [app name]who are not necessarily like struggling with mental health issues. I think it’s much less stigma around that than like going to an appointment.”* [Participant 17] |
|  | ***5.2 Loyalty and reliance*** | *“People can be good, people can be listened to but you just do not know what to expect from people even if you ask them a, not everyone, whereas if you are relying on an app you know that there’s an engagement, there’s a commitment, where you are continuing, you’re continuing so erm you feel more connected and you feel more engaged in the long term. Like there's no sense of like guilt like it's going to come to an end soon, or you know I don't want to talk to you about this or there is no issues or we're working towards it. The negativity around recovery around and journey, your own journey is supported and facilitated by using the app than compared to an actual person.”* [Participant 15]  *“I know I can rely on it now, the app is always there for you.”* [Participant 11]  *“You know, there's an element of trust isn't there. I guess an app is always there and I am not going to feel guilty about contacting an app.”* [Participant 06]  *“Suppose in a way apps are almost more reliable than a person. And because it's there, and if your device breaks or whatever, you just get another one and download it again. Like it's there for you, there's always a way to access it and you can't not have it really like. So if you need it, you can access it whenever you want, whereas you can't do that with a person and. And yeah obviously when you have counselling or therapy, you know, you know we either get given a set amount of sessions, or even if you don't it will come to an end at some point um. And you can't just find someone and have access to them whenever you feel like it, and so yeah.”* [Participant 16]  *“…basically services will help you get past the like the severe dip and then basically it's it stops, there's no ongoing support. And that kind of does make me feel stranded.”* [Participant 01]  *“You can't move until you haven't completed each section, so there is a level of trust, there is a level of respect.”* [Participant 05] |
