# Supplementary material for: A qualitative study exploring the digital therapeutic alliance with fully automated smartphone apps
Source: Digit Health. 2024 Dec 15;10:20552076241277712. doi: 10.1177/20552076241277712 (PMC11648025; doi:10.1177/20552076241277712)
Supplement: sj-docx-2-dhj-10.1177_20552076241277712 - Supplemental material for A qualitative study exploring the digital therapeutic alliance with fully automated smartphone apps [file sj-docx-2-dhj-10.1177_20552076241277712.docx]

**Demographic questionnaire**

| **Participant code** (to be completed by researcher) |  |
| --- | --- |

| **Date** |  |
| --- | --- |

As discussed, we would like to gather some more information about you. You do not have to complete any item you do not wish to. Please ask if you have any questions.

| **DOB:** | |
| --- | --- |
| **Gender:**  Do you identify as:   1. Male 2. Female 3. Transgender 4. Non-binary 5. Prefer rather not say 6. Other- please state: | **Ethnicity:**  Do you identify as:   1. White/Caucasian 2. Mixed/Multiple Ethnic Groups 3. Asian/Asian British- Bangladeshi 4. Black/African/Caribbean/Black British 5. Arabic 6. Prefer rather not say 7. Other – please state: |
| **Marital status:**   1. Single 2. Co-habiting 3. Married 4. Divorced 5. Widowed 6. Prefer not to say | **Employment status:**   1. Employed 2. Self-employed 3. Out of work and looking for work 4. Out of work but not looking for work 5. Student- PhD 6. Retired 7. Unable to work 8. Prefer not to say |
| **Highest completed level of education:**   1. Primary school 2. Secondary school 3. Diploma or equivalent 4. Trade/technical/vocational training 5. University bachelor’s degree 6. University master’s degree 7. PhD or higher 8. Prefer not to say | **Mental health difficultly:**  Do you identify as experiencing:   1. Low mood/depression- Main Dx 2. Anxiety – a little bit 3. Psychosis 4. Personality difficulties 5. Other- please state: |
| **Living situation:**   1. Living alone 2. Living with partner 3. Living with parents 4. Living with relatives 5. Living with others e.g. friends, other 6. Prefer not to say | **Do you currently (or in the last six months) have a diagnosis of a mental health difficulty?**   1. Yes 2. No 3. Unsure   If yes, please provide details:  …………………………………………….  …………………………………………….  ……………………………………………. |
| **Are you currently under a mental health service?**   1. Yes 2. No 3. Unsure   If yes, please provide details:  …………………………………………….  …………………………………………….  ……………………………………………. | **Are you currently taking medication for your mental health?**   1. Yes 2. No 3. Unsure   If yes, please provide details:  …………………………………………….  …………………………………………….  ……………………………………………. |
| **Are you currently receiving any psychological therapy?**   1. Yes 2. No 3. Unsure   If yes, please provide details:  …………………………………………….  …………………………………………….  ……………………………………………. |  |
| **APP USE**  **What is the name of the app/apps you use?**  **Where did you download the app from (i.e. apple/android app store)?**  **Main function of the app?**  **Main reason for using the app?**  **How long have you been using the app?**  **How often are you using the app?** | |
